# Understanding the Barriers Fathers Face to Seeking Help for Paternal Perinatal Depression: Comparing Fathers to Men Outside the Perinatal Period

**DOI:** 10.3390/ijerph21010016

**Published:** 2023-12-21

**Authors:** Megan Reay, Andrew Mayers, Rebecca Knowles-Bevis, Matthew T. D. Knight

**Affiliations:** 1 Oxford Health NHS Foundation Trust, The University of Oxford, Oxford OX4 4XN, UK; rebecca@psychologyoxfordshire.co.uk (R.K.-B.); matthew.knight@hmc.ox.ac.uk (M.T.D.K.); 2 Department of Psychology, Bournemouth University, Poole BH12 5BB, UK; amayers@bournemouth.ac.uk

**Keywords:** perinatal mental health, fathers, postnatal depression, men’s mental health

## Abstract

Research has shown that men are less likely than women to seek help for depression at any time of life due to barriers, including stereotypical masculine norms and stigma. The evidence suggests that approximately 10% of fathers experience postnatal depression, yet new and expectant fathers are not routinely offered screening or support in the same way as mothers. Therefore, this research explored the barriers fathers face to seeking help for paternal perinatal depression (PPD). Data were collected using an online survey. Initially, fathers with postnatal depression were compared to men experiencing depression at another time of their life in terms of their attitudes to seeking psychological help, conformity to masculine norms, self-stigma, and awareness of services. Secondly, a proposed model of help-seeking amongst fathers with postnatal depression was evaluated. Finally, additional barriers to help-seeking for paternal postnatal depression were explored qualitatively. A total of 125 participants took part in the quantitative comparison, and 50 of the fathers also provided qualitative data. No between-group differences were found, suggesting that the existing literature on barriers to seeking help for male depression is applicable to fathers with postnatal depression. The qualitative results also highlighted the need for better awareness of paternal postnatal depression and better access to services for fathers. Limitations, implications for policy, and directions for future research are discussed.

## 1. Introduction

### 1.1. Paternal Postnatal Depression

The Diagnostic and Statistical Manual (5th edition) (DSM-5) criteria for perinatal depression capture depression with an antenatal onset, as well as depression which begins postnatally [1]. However, the majority of the research on which these criteria are based concerns mothers but not fathers. A meta-analysis of 43 papers indicated that approximately 10% of men experience depression following the birth of their child, which is twice the rate of depression amongst men of the same age in the general population [2,3]. The prevalence of depression rises to 25–50% in men whose partner also experiences postnatal depression [4]. Symptoms of postnatal depression include low mood, a lack of interest in activities, feeling anxious, guilty and worthless, as well as possible self-harm occurring during the two years after the birth of a child [5]. Fathers are also known to express more aggression, irritability and anxiety than mothers [6]. This has led researchers to suggest that fathers may experience a different condition known as paternal perinatal depression (PPD) [6].

PPD is a key concern for public health, as it has significant implications, not just for the fathers themselves but also for their partners and children. PPD has been associated with higher levels of marital dissatisfaction, as well as increased emotional and behavioural problems in children [7,8]. In the UK, it is now recommended that fathers be included throughout antenatal care and that all fathers be screened postnatally for depression [9]. In practice, though, new fathers in the UK are rarely offered support from specialist perinatal mental health services. Fathers and healthcare professionals report barriers that include a lack of awareness, stigma, and policies which prioritise access for mothers [10]. As a result, many fathers do not access the mental health support they require.

### 1.2. Father’s Psychological Help-Seeking

Despite the growing literature on PPD, there is limited research specifically looking at potential barriers to fathers seeking help. Men are believed to be less likely than women to seek help for any health condition, including depression [11]. 

Male psychological help-seeking is predicted by levels of conformity to masculine norms and self-stigma [12]. Research considers traditional masculine norms to include factors such as competition, violence and self-reliance [13]. Self-stigma is a subtype of stigma which occurs when negative attitudes are internalised [14]. Self-stigma has been shown to have the largest direct effect size on help-seeking and to partially mediate the relationship between conformity to masculine norms and help-seeking [12,15,16]. It may act as a barrier to help-seeking, as it is known to lower self-esteem and reduce motivation to achieve behavioural goals [17].

In their review of the literature, Latalova and colleagues (2014) propose a model of male help-seeking for depression in which they hypothesise that willingness to seek psychological help is directly predicted by men’s attitudes towards counselling and that those attitudes are directly predicted by self-stigma and masculine norms [18]. Masculine norms and depression also predict attitudes indirectly through their impact on self-stigma. This model closely mirrors the Theory of Planned Behaviour (TPB) [19], which suggests that a behaviour such as help-seeking is predicted by one’s intention to complete the behaviour. A person’s intention is predicted by their attitudes towards a behaviour, their group’s subjective norms and their perceived control over whether they can execute the behaviour.

There is only very limited research exploring help-seeking amongst fathers experiencing mental health difficulties during the postnatal period. Qualitative interviews with fathers identify similar barriers to those experienced by men outside of the perinatal period, including stigma and notions of masculinity [20,21,22]. However, fathers also report experiences that are specific to the perinatal period, such as services being centred around mothers, and finding that taking on the role of a ‘father’ conflicted with their previous ideas of masculinity [20]. As well as stigma and masculine norms, fathers also cite exclusion from services and a lack of awareness of postnatal depression or available help as further barriers [20,21,22]. This suggests that fathers with PPD may face additional barriers to help-seeking compared to men with depression occurring at another time of life. However, it has not yet been established whether these additional barriers result in lower help-seeking intentions amongst fathers with PPD.

In the UK, perinatal services provided by the National Health Service (NHS) are beginning to screen fathers for PPD, whereas previously, support was only available to mothers. The NHS definition of the perinatal period has also been extended to include pregnancy and 24 months postpartum [9]. This study provides an updated account of the barriers fathers face to seeking help during this time period, which will support perinatal services in the UK in the development of their services for fathers with PPD.

### 1.3. Current Research

The primary goal of the current research was to understand whether fathers face increased barriers to seeking help for PPD compared to men experiencing depression outside the perinatal period and whether these barriers result in fathers exhibiting lower help-seeking intentions compared to men outside of the perinatal period. To meet this goal, levels of known barriers and help-seeking intentions of fathers with PPD were compared to men with depression occurring at another time of life. The specific primary hypotheses were as follows:

- Fathers with PPD will show lower help-seeking intentions compared to men with depression occurring at another time of life.
- Compared to men with depression occurring at another time of life, fathers with PPD will show:
- Higher self-stigma;
- Higher conformity to masculine norms;
- Lower awareness of support services.

**Figure 1:**
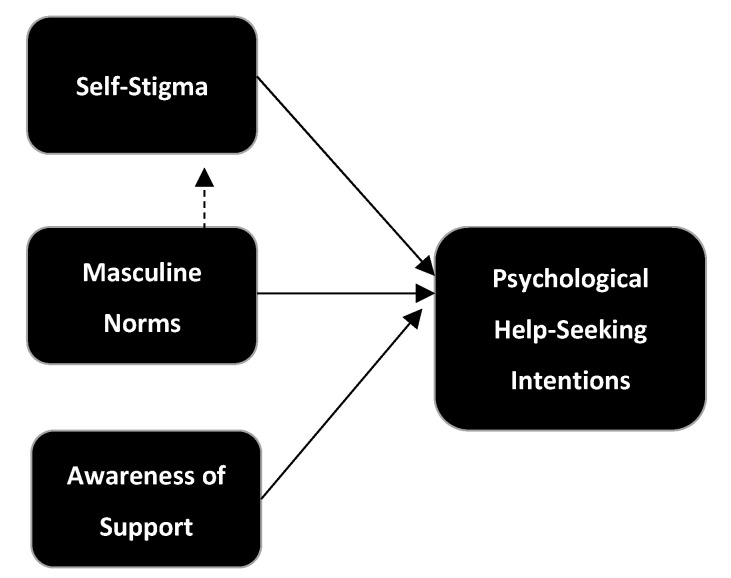
Proposed model of help-seeking amongst men with PPD.

The secondary goal of this research was to conduct an exploratory analysis to evaluate a proposed model of help-seeking for PPD (see Figure 1). This proposed model combined the TPB with Latalova et al.’s (2014) model: self-stigma maps onto the attitudes described in TPB, and masculine norms relate to subjective norms from TPB. The proposed model also integrates findings that self-stigma partially mediates the link between masculine norms and help-seeking intentions [23]. It also includes a third variable related to perceived behavioural control from TPB named “awareness of support”, which is supported by the qualitative literature on fathers with PPD [20,22]. As specialist perinatal mental health services in the UK do not routinely offer psychological support to fathers, the current research uses help-seeking intentions, rather than help-seeking behaviour, as the primary outcome.

This research also considered several additional potential barriers for which there is as-yet insufficient research upon which to base predictions regarding their influence on help-seeking intentions. These were: partner’s depression status, current severity of depression, depression history, and history of help-seeking. Data on ethnicity, age and number of previous children were also collected, as these have been shown to impact help-seeking for male depression [23,24,25].

The specific hypotheses In relation to the secondary analysis of the proposed model were as follows:

- Higher self-stigma will lead to lower help-seeking intentions, independently of the contributions made by masculine norms and awareness of support;
- Self-stigma will partially mediate the relationship between masculine norms and help-seeking intentions.
- Fathers’ help-seeking intentions will be predicted by:
- Severity of depression;
- Previous history of depression;
- History of help-seeking;
- Partner’s depression.

In addition, the qualitative component of the study aimed to understand the barriers to fathers seeking psychological help for PPD. These barriers for PPD were explored qualitatively using reflexive thematic analysis to allow fathers to share details of their experiences seeking help and recommendations on how this could have been improved [26,27]. The research aimed to explore the following question:

- What are the perceived barriers to help-seeking amongst fathers with paternal perinatal depression?

## 2. Materials and Methods

### 2.1. Design

The research used a cross-sectional between-groups design in which all the data were gathered online at one time point. The research compared fathers who had experienced PPD during the two years after their child was born with men who had experienced depression during the past two years but had not had a child during this time and, therefore, were not in the perinatal period. Variables were naturally occurring; no experimental manipulation was involved. All data were collected online to allow participants to take part anonymously, as it was hoped that this would make the research more accessible to some fathers who may not have otherwise taken part due to stigma or shame related to PPD.

The men who had experienced PPD in the past 24 months were also invited to complete a series of free text responses to questions. An expert by experience (EbE) was consulted when developing the research questions and questionnaires. The EbE was somebody with lived experience of PPD who now campaigns for improved mental support for fathers, and he had previously supported the authors in completing research in this area. He was involved particularly in supporting the wording of the qualitative questions and providing support and ideas for recruitment. A key recommendation from our EbE was to not include the term ‘postnatal depression’, as they felt this is so closely associated with mothers that it may discourage participation from fathers.

### 2.2. Participants

The two groups compared in the research were the father depression group (FD) and the male depression group (MD). The father depression (FD) group consisted of fathers who had experienced PPD following the birth of their child in the past 24 months. The male depression (MD) group consisted of men who had experienced depression within the past 24 months but who had not had a child during this time, and their depression was therefore not considered to be PPD. Participants in the MD group could be fathers but were not currently in the perinatal period, as their children were born more than 24 months ago. This research defined the perinatal period as up to 24 months after the birth of a child to align with the NHS long-term plan to expand mental health support in the perinatal period up to 24 months postpartum [9].

All participants had to meet the following criteria:

- Identifies as male;
- Has experienced self-reported depression in the past 24 months;
- Between 18–65 years of age.
- In addition, the FD group were required to meet the following:
- In the perinatal period (partner has given birth in the past 24 months);
- Biological fathers (i.e., not adoptive fathers or step-fathers).
- The exclusion criteria for all participants were as follows:
- No access to suitable technology to complete the study;
- Self-report of a history of serious mental illness. Following advice from experts by experience, no specific diagnostic labels were used as exclusion criteria, instead, participants could determine for themselves if they had a history of a serious mental illness.

### 2.3. Procedure

Data were collected online using Qualtrics between April and December 2021. Participants were recruited using electronic posters containing details of the project and URL and QR codes to access the survey. Electronic posters were shared across targeted social media platforms by researchers, campaigners and charity organisations, including the Fatherhood Institute, Andy’s Man Club and The Dad Pad. Efforts were made to recruit a diverse population by contacting support groups for fathers from minority populations and requesting their support with recruitment.

Prior to accessing the survey, potential participants read an information sheet containing details of the research before they could give informed consent to take part. To ensure that potential participants were eligible to take part in the research, they were asked to confirm that they had experienced a list of specific symptoms of depression, which were taken from the DSM-5 [1], as well as male-specific symptoms, such as agitation or irritability [6]. Participants who reported that they did not meet the DSM-5 criteria for depression were not eligible to take part and were removed from the study.

All eligible participants then completed a series of quantitative measures electronically, which are described below. After completing the quantitative measures, participants in the FD group were also invited to respond to five open questions. The FD group were asked (1) whether they had experienced any barriers to seeking help for paternal perinatal depression and, if so, (2) what these barriers were, (3) what help they would have liked to be available, (4) how they could have been supported to access this, and (5) if they had any further comments.

Upon completion of the survey, participants were directed to an electronic debrief page. This contained contact details for the researchers, as well as details of organisations that offer support to men struggling with their mental health.

### 2.4. Measures

The following measures were completed online using the Qualtrics platform. None of the items used forced-choice response, meaning that participants could choose not to complete certain measures or items within measures.

Demographics: The participants initially completed a demographic questionnaire, which captured age, ethnicity and number of previous children.

Help-seeking: Participants’ help-seeking intentions were measured using the Attitudes Towards Seeking Professional Psychological Help Scale short form (ATSPPH-SF) [28]. The ATSPPH-SF a ten-item measure rated on a 4-point scale. The ATSPPH-SF is specific to psychological help-seeking and includes three dimensions: openness to seeking professional help, perceived value in seeking professional help, and preference to cope on one’s own [29]. The scale has good internal consistency (α = 0.78) and construct validity [30]. Higher scores reflect positive intentions to seek help.

Participants were also asked to select ‘yes’ or ‘no’ to indicate if they were currently accessing any form of support for depression.

Depression: Depression was measured using the Depression, Anxiety and Stress Scale (DASS-21) [31], a shortened version of the original 42-item measure. The 21-item version is rated on a 4-point scale (0–3). Both the 42- and 21-item versions show good reliability (α = 0.93) and validity [32]. Total scores of 60 or above out of 126 are considered high and indicative of more severe depression. The DASS-21 contains depression, anxiety and stress subscales and was selected, as PPD includes features of stress and anxiety, as well as traditional symptoms of depression [6].

Participants were then asked whether they had previously experienced depression, if they had previously sought help for depression, and whether their partner (if applicable) was currently experiencing depression.

Self-stigma: Self-stigma was measured using the Self-Stigma of Seeking Help Scale (SSSHS) [33], a 10-item questionnaire with good reliability (α = 0.83) and construct validity across different cultures [34]. Responses are rated on a 5-point scale (1–5), with higher scores indicating higher self-stigma.

Masculine Norms: The Conformity to Masculine Norms Inventory-46 (CMNI-46) was used to measure levels of traditionally masculine values. This is a brief, 46-item version of the original scale, which is scored on a 4-point scale (CMNI) [35]. The CMNI-46 accurately measures nine of the original 11 subscales (Winning, Emotional Control, Primacy of Work, Risk-Taking, Violence, Heterosexual Self-Presentation, Playboy, Self-Reliance, Power Over Women) [36]. The CMNI-46 also shows good internal reliability (median α = 0.82) and strong convergent and divergent validity [31].

Awareness of services: Participants reviewed a checklist of nine support services and indicated which they were familiar with, as well as being asked to list any additional services they were aware of. These were totalled together to give a numerical value for awareness of services.

### 2.5. Analysis

Due to a lack of existing literature exploring help-seeking intentions in fathers, it was not possible to use established effect sizes to determine the number of participants needed to power the between-subjects comparison. Therefore, effect sizes from similar papers were used to estimate the effect size. One paper compared help-seeking between people with depression and people without depression and found an effect size of d = 0.5 [37]. Another paper used the Attitudes Towards Seeking Professional Psychological Help Scale (ATSPPH) and compared help-seeking intentions between heterosexual and homosexual men with depression. They also reported an effect size of 0.5 [12]. Therefore, a medium effect size (d = 0.5) was assumed for power calculations, which were completed using G*Power software (v.3.1.9.7). In order to adequately power the primary hypothesis regarding between-group differences in help-seeking intentions with a 0.05 significance level, at least 102 participants were required (*n* = 51 in each group).

Prior to completing any statistical analysis, the relevant assumptions were checked. In order to better define and understand the groups, initially, the demographic data for the FD and MD groups were statistically compared using chi-squared analysis, Fisher’s exact test or their non-parametric equivalent. The main analysis involved completing a series of independent sample t-tests to statistically compare the groups in terms of their help-seeking intentions, levels of self-stigma, conformity to masculine norms and awareness of services. A hierarchical regression model was then used to test hypotheses 3, 4 and 5. The planned mediation analysis from hypothesis 4 was conducted using the Baron and Kenny [38] approach to mediation. All statistical analyses were completed using the SPSS-27 statistical programme.

Qualitative data from fathers were analysed using reflexive thematic analysis [27]. The epistemological and ontological approach taken to the reflexive thematic analysis was contextualism and critical realism. Authors looked to find a level of truth or reality in respect to the barriers described by fathers whilst acknowledging that fathers’ responses and the data analyses were influenced by the context and culture in which they were studied. Reflexive thematic analysis was used as the approach is suited to the above theoretical positioning. Additionally, the method’s flexibility allowed for the analysis of survey responses, which are often briefer than other qualitative data [26,27]. An inductive approach was taken to coding, as the authors aimed to understand fathers’ experiences without being influenced by previous research or theory. A reflexive diary was kept throughout the analysis to support authors to identify biases brought to the interpretation and analyses.

## 3. Results

### 3.1. Participants

A total of 125 participants took part in the research: 64 men in the perinatal period (FD) and 61 men outside of the perinatal period (MD). Due to attrition, some participants did not complete all of the quantitative measures, which resulted in differing numbers of responses for each measure.

As can be seen in Table 1, there were no significant differences between the FD and MD groups in terms of their ethnicity. The groups did differ significantly in terms of age; the FD group was significantly older than the MD group (*p* < 0.001). The groups also differed in terms of fatherhood status, with significantly more participants in the FD group being fathers (*n* = 64) compared to the MD group (*n* = 18) (*p* < 0.001). The number of years since the birth of the youngest child in the MD group ranged from 2.79 years to 31.36 years, with a mean of 9.85 years.

### 3.2. Comparing Fathers (FD) to Men with Depression Outside of the Perinatal Period (MD)

Means and standard deviations for between-group comparisons are given in Table 2. In relation to our primary hypothesis, an independent samples t-test comparing the FD and MD groups found no significant difference in help-seeking intentions as measured by the ATSPPH-SF (t = −0.46, *p* = 0.65).

Similarly, no significant differences between the FD and MD groups were found for levels of self-stigma (t = 1.09, *p* = 0.27) or conformity to masculine norms (t = −1.63, *p* = 0.11). Due to the data not being normally distributed, a non-parametric Mann–Whitney U test was used to compare awareness of services between the FD and MD groups, which again identified no significant between-group difference (U = 1293, z = −1.334, *p* = 0.18). There were also no significant differences between the groups in terms of their current depression levels, history of depression, partner’s current depression, history of help-seeking or current help-seeking.

### 3.3. Evaluating a Proposed Model of Help-Seeking

The secondary hypotheses (3–6) related to exploring a proposed model of help-seeking amongst fathers. Prior to beginning the planned regression analysis, all statistical assumptions were checked. Our analysis identified that all assumptions were met, as there was a linear relationship between the predictor variables and the dependent variables. There was also homoscedasticity for the residuals, no significant outliers and the residuals were normally distributed. Additionally, the correlation coefficients between each predictor variable were all <0.7, and all tolerance values were >0.1, showing that there was no multicollinearity between variables.

A hierarchical regression model was then constructed using the FD data in a step-wise fashion (*n* = 53). Initially, only two predictor variables, conformity to masculine norms and awareness of services, were added to the model. The third hypothesis was accepted as adding self-stigma to the model, alongside conformity to masculine norms and awareness of services, significantly increased the amount of variance in help-seeking intentions that were accounted for (R^2^ = 0.19, *p* < 0.001). The overall model with all three predictors was significant (F[3, 49] = 3.88, *p* = 0.02).

This regression model was then used to explore whether self-stigma mediated the relationship between conformity to masculine norms and help-seeking intentions. The first stage of Baron and Kenny’s [38] approach to mediation analysis involved exploring whether masculine norms significantly predicted help-seeking intentions, which was not the case (standardized β = −0.23, *p* = 0.09). However, the requirement for this relationship to be statistically significant has been heavily criticised, therefore, we followed the approach of more recent publications [39] and proceeded with the planned analysis. The results of the remainder of the planned mediation analysis identified that conformity to masculine norms did significantly predict self-stigma (standardised β = 0.30, *p* = 0.03) and the full model, with both self-stigma and conformity to masculine norms as predictors, was significant (*p* = 0.00). Additionally, the observed standardised beta values for the relationship between conformity to masculine norms and help-seeking intentions decreased with the addition of self-stigma from −0.23 to −0.11 (standardised β = −0.11, *p* = 0.42). These results suggest that self-stigma does partially mediate the relationship between conformity to masculine norms and help-seeking intentions and that a large proportion of the variance in father’s help-seeking intentions, which was thought to be explained by men’s conformity to masculine norms, is better explained by their levels of self-stigma.

**Figure 2:**
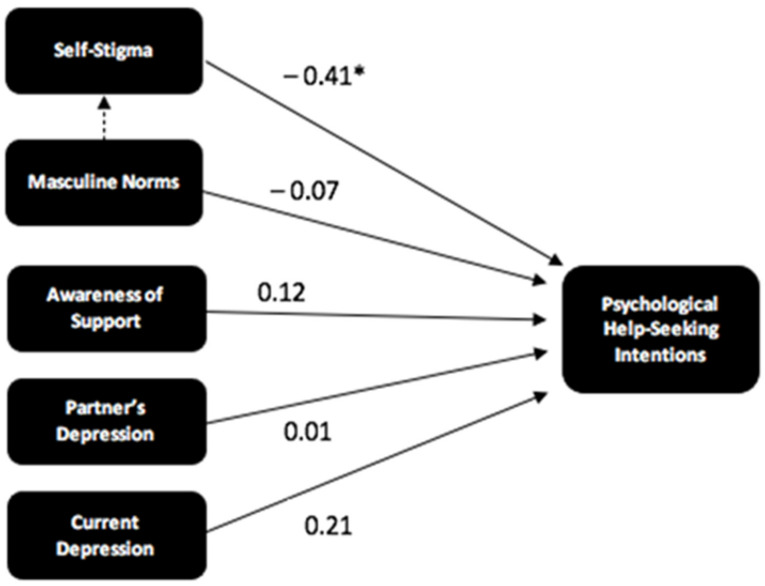
Model of psychological help-seeking intentions amongst fathers. Standardised beta values are shown. * indicates a significant predictor variable (*p* < 0.05). A dashed line indicates a mediation effect.

The additional exploratory predictor variables of partner’s depression, severity of current depression, depression history, and history of help-seeking were then added to the regression model (*n* = 52), see Table 3 Including current partner’s depression led to a very small increase in the total amount of variance explained from R^2^ = 0.183 to R^2^ = 0.184. Adding participants’ levels of current depression further increased the amount of variance explained to R^2^ = 0.23. The addition of the other two variables did not increase the total amount of variance explained and were, therefore, not included in the final model. The final model was statistically significant (F[5, 46] = 2.65, *p* = 0.04) and is depicted in Figure 2, including the standardised beta values for each relationship.

### 3.4. Understanding the Barriers to Seeking Help for Paternal Perinatal Depression

**Figure 3:**
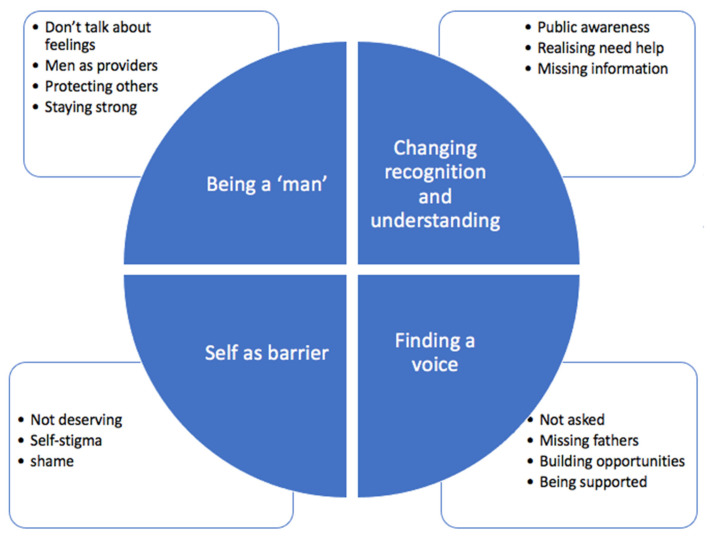
Summary of the four themes and fourteen subthemes created following reflective thematic analysis.

Reflexive thematic analysis was used to generate four themes (consisting of 14 sub-themes), which are depicted in Figure 3 and described below [27].

#### 3.4.1. Being a ‘Man’

The first theme described barriers to seeking help for paternal perinatal depression in terms of societal pressure on fathers to act in ways that are considered culturally and socially appropriate for men.

Do not talk about feelings: Fathers described pressure to not discuss their feelings. Fathers spoke of a “*lack of practice*” [WH13] discussing emotions as “*men are not taught to tune into their emotional side*” [BI11]. One shared that a “*lack of experience discussing feelings, and a sense that I’d be thought of as just making a fuss about nothing*” [ST01] was a barrier to seeking help. This suggests that, even if the fathers had sought help, they may have struggled to discuss their emotional experiences.

Men as providers: This subtheme described fathers prioritising providing for their family over attending to their own well-being: “*there is very little time when supporting a newborn and wife to focus on yourself*” [LO04]. Men described “*needing to keep going to work to support my family”* [FA08] and facing institutional barriers, such as workplaces offering “*no time off to do so [seek-help]*” [WY06].

Protecting others: Another barrier was the perceived societal pressure to protect others, resulting in concerns about becoming a “*burden*” [BI24]. Fathers described “*not wanting to overshadow my partner’s needs”* [BE17] and feeling “*selfish*” [SO20] for sharing their own struggles with low mood. Fathers worried about their partners as they had “*actually had to give birth and have the physical stress of birth. I feel like surely my issues can’t be as serious as that of the mother*” [SO4].

Staying strong: The final subtheme described societal expectations to appear “*strong for family and not wanting to show weakness to friends*” [ED20]. Fathers felt they should be strong, “*as a dad, I didn’t want to show weakness*” [QU09].

There was a sense that had fathers wanted to break these stereotypes and seek support for mental health difficulties, they may have struggled due to lack of practice, societal pressure and institutional barriers.

#### 3.4.2. Changing Recognition and Understanding

The second theme spoke to the need to increase recognition and understanding of paternal perinatal depression to enable fathers to seek help. Fathers described a lack of public and professional knowledge, making it hard to recognise the signs of paternal perinatal depression or to access information and support.

Public awareness: Participants described a lack of public and professional awareness of paternal perinatal depression as a barrier to seeking help. One father reported that he could not access support for paternal perinatal depression as he “*never knew it existed”* [BI24], and another felt that the public held a “*view that it’s not something that affects men*” [BO17]. Fathers also felt that “*there’s no awareness of GPs and other professionals for it”* [SI02], which prevented men from seeking help as they worried about whether paternal perinatal depression “*would be taken seriously*” [ST01] by professionals.

Realising need for help: Due to this lack of awareness, men reported that “*realising that you might need help is hard*” [BI11]. Others struggled to recognise the signs of paternal perinatal depression as the concept “*just doesn’t connect with me*” [MS01]. This made it challenging for fathers to recognise they were experiencing paternal perinatal depression and “*to accept that I needed it [help]*” [OP14].

Missing information: Fathers described the difficulties they faced when searching for information about paternal perinatal depression. Men described not knowing “*how to go about getting help, or what help I needed*” [WH18]. Fathers were not “*made aware of services by the midwife or health supporter*” [RO21] and struggled with a “*lack of available or clearly advertised services*” [BE17]. One father reported “*there’s no reference to it on Google’s first hits. There’s a lot on women scenarios and focus that makes me feel uncomfortable even to suggest paternal depression*” [SI02]. This lack of information and understanding hindered fathers from recognising PPD or knowing how to seek support.

#### 3.4.3. Finding a Voice

This theme represented the barriers fathers faced at each stage of being heard and supported by professionals, peers and family members.

Not asked: The first subtheme related to fathers “*not being asked”* [BE17] about their wellbeing by professionals. Fathers felt that “*it’s [paternal perinatal depression] just not spoken about and I was never asked*” [MS01]. Fathers felt that “*any kind of engagement post birth to check in on my well-being*” [WO16] would have been beneficial and advocated for “*at least one occasion on which a medical professional talks to the father alone about their experiences and needs*” [FI08].

Missing fathers: Fathers shared experiences of specialist services being unavailable to them as the “*system is geared to mothers, not fathers*” [ED20]. Many described the “*lack of dedicated paternal depression resources”* [MC08] and shared that *“there should have been someone there and there wasn’t*” [BI24]. This was compounded by COVID-19, with one father saying “*I think due to the pandemic and not being able to attend anything fathers should be getting more support and they’re not. It’s all about the mum but the dad needs to get help too*” [MC07], and another described becoming a “*passenger*” [SO4] in the process.

Building opportunities: Participants indicated they would have benefitted from opportunities to form a “*community of people with similar experiences*” [MC08] through counselling or support groups. This felt important in creating a safe space for fathers to discuss their experiences. Men called for “*a male friend to talk to*” [CL10] and more professional input to access “*group therapy*” [LA20], “*phone-based talking therapies*” [BE17] or “*self-help guides*” [WH13].

Being supported: The final subtheme spoke to the sub-group of men who did find a voice and sought help, which was “*just right*” [WH18]. Although some fathers felt well supported, they reported that this help came typically from family or friends rather than from professionals. When asked about barriers to speaking with family and friends, the majority of fathers described the barriers highlighted in the other subthemes. However, approximately one-fifth of fathers felt “*there were none, my family and wife were great*” [MC07]. This suggests that existing networks could be a useful resource for fathers to access the support they need whilst barriers to accessing professional input remain in place.

#### 3.4.4. Self as a Barrier

The final theme related to men frequently referencing themselves as a barrier due to negative beliefs and feelings about experiencing PPD creating high levels of self-stigma.

Not deserving: Several fathers described not seeking help for PPD, as they felt that they did not deserve help in the same way as mothers or other men experiencing depression outside of the perinatal period (“*my form of depression wasn’t worthy*” [ON5]). Others worried about not being “*depressed enough*” [BR27] to deserve help, with one father stating “*I didn’t think it was bad enough and I didn’t want to take the service away from someone who needed it more*” [WH18].

Self-stigma: Fathers shared self-stigmatising attitudes about PPD and how this prevented them from seeking help. Fathers described the “*stigma of a man having paternal depression [and] thinking this should be happiest time of my life*” [ON5], with one man reporting, “*I was my biggest barrier”* [MC08]. Others felt “*scared of what people will say*” [WI04] if they were to seek help. These internalised negative attitudes prevented fathers from seeking help.

Shame: The final subtheme described the emotional barriers associated with self-stigma. Fathers felt they “*should be enjoying my newborn and not feeling down, so the shame and guilt that comes with that*” [SE14]. Others stated that “*shame prevented me from talking to friends*” [ES11]. This theme highlights that, in addition to the societal, institutional and practical barriers men face to seeking help for PPD, they must also overcome their own internal barriers.

## 4. Discussion

To our knowledge, this paper is the first empirical study of help-seeking amongst fathers with postnatal depression. The results indicate that fathers with PPD are not significantly different to men with depression occurring at another time of life in terms of their attitudes to help-seeking, self-stigma, conformity to masculine norms or awareness of services. The proposed model of help-seeking amongst fathers was explored and supported by the current findings. Qualitative responses also highlighted additional societal, institutional and practical barriers fathers face to seeking help for postnatal depression.

This research found no significant differences between fathers and men outside of the perinatal period in terms of self-stigma, conformity to masculine norms, level of depression or help-seeking intentions. This result is also corroborated by the regression analysis, as it supports the proposed model of help-seeking amongst fathers, which is in line with existing literature on male help-seeking outside of the perinatal period [18]. As is also seen in men outside of the perinatal period, this research found that for fathers, self-stigma explained the largest amount of variance in help-seeking intentions. Further, self-stigma partially mediated the relationship between conformity to masculine norms and help-seeking intentions [12,18]. Therefore, reducing self-stigma is vital to reducing barriers to help-seeking amongst fathers. As self-stigma is a process whereby public stigmatising attitudes are internalised [40], interventions at the public health level may be most effective, such as increasing the visibility of men experiencing PPD, educating the public about PPD, and working to reduce stereotypical masculine attitudes. Additionally, work to support fathers with PPD should aim to empower fathers and challenge internalised stigma. Future research could explore the efficacy of such interventions.

A difference between help-seeking amongst fathers compared to men outside of the perinatal period is that, unlike in previous research with men [12], conformity to masculine norms did not significantly predict help-seeking for fathers in the perinatal period. This suggests that fatherhood may reduce men’s levels of conformity to masculine norms. This is in line with Addis et al.’s proposal that conformity to masculine ideals varies as a result of context [41]. However, no differences were found in the present study in rates of masculine norms between fathers with PPD and men with depression at another time of life. Existing research suggests that masculine norms are instilled at a young age and remain stable over time, even during significant life transitions [12,30]. More research is required to understand the role of masculine norms in male help-seeking intentions and whether conformity to these norms is altered during the perinatal period.

This research also identified that a lack of public understanding of PPD and exclusion from specialist services are key barriers to fathers seeking help for PPD. The inclusion of ‘awareness of services’ as a barrier to help-seeking amongst fathers was supported by the quantitative model and reported frequently in the qualitative responses. These barriers are not reported in previous quantitative work regarding seeking help for male depression, which may suggest they are more specific to men during the perinatal period [18]. This is consistent with previous qualitative research with fathers who reported struggling to access specialist services due to society not understanding paternal perinatal depression and feeling services only supported mothers [20,21]. Future research could build upon these results by developing interventions to target these barriers and explore the impact of such interventions using validated quantitative measures.

The results clearly highlight the importance of perinatal mental health services including fathers in their mental health screening processes. Fathers described never being asked about their mental health. This serves as a barrier at multiple levels; it means that services are simply not made available to fathers, and it communicates to fathers that their own well-being is less important than the mother’s. This perpetuates the masculine stereotypes that fathers described in the ‘being a man’ theme. It is possible that if fathers were routinely asked about PPD, this would also support raising awareness and knowledge of PPD amongst the public and professionals.

### Limitations

One limitation of this research was the modest sample size. The sample may have been too small to detect smaller effect sizes, as the power analysis was based primarily on the between-group comparison rather than the exploratory regression analyses. Future research is needed to explore the proposed model with larger sample sizes. However, fathers with PPD are challenging to recruit to research of this kind for reasons including high self-stigma and a lack of awareness. Future researchers could overcome barriers to research participation by offering paid incentives or simplifying recruitment processes [42]. Alternatively, as some small effect sizes may not be meaningful clinically, future research may instead choose to utilise qualitative methodologies to gain a detailed, clinically useful understanding of barriers to help-seeking, as this would not require such a large sample.

As well as being relatively small, the sample lacked adequate diversity, as the majority of participants identified as White British. Therefore, this research underrepresented fathers from other ethnic backgrounds. It is possible that fathers with PPD from ethnic minority backgrounds experience even higher rates of stigma than their White counterparts. This phenomenon is referred to as double stigma [43] and could mean that those from ethnic minority backgrounds were less likely to take part in the research or to seek support for PPD. Therefore, recruiting a predominantly White British sample may have missed the additional barriers to help-seeking experienced by fathers facing this double stigma. Future research must focus on recruiting fathers from minority populations to enable policies and treatments for paternal perinatal depression to be relevant to all fathers.

A further limitation is that comprehensive and objective data on participants’ perinatal and mental health history were not collected. The majority of participants in both groups had previously experienced depression, but it is unclear how long their recent episode of depression lasted, as no chronicity data were collected. Therefore, it is possible that some participants in the MD group were experiencing depression, which began in the perinatal period over 24 months ago. Whilst this would no longer be considered PPD, it poses a challenge to drawing firm conclusions about the lack of between-group differences. Additionally, the exclusion criteria of a self-rated history of serious mental illness were subjectively assessed, meaning that some potential participants with a history of depression may have been missed if they considered this a serious mental illness. It is also possible that men with depression who also experienced high levels of self-stigma would not identify as having depression and, therefore, would not have been included in the study. There were also no data collected on their partner’s pregnancy, birth, or their child’s development, all of which may have had an impact on the father’s mental health. Therefore, future research should collect more comprehensive data, including data on chronicity of depression and perinatal history, as well as introducing more rigorous exclusion criteria regarding fatherhood status and mental health history to allow for clearer conclusions.

This research gathered qualitative data online using free text boxes as part of a larger, mixed-methods study. This design was selected to allow fathers to take part in the research anonymously, following advice from our expert by experience that shame or stigma may prevent fathers from wishing to take part in less anonymous forms of research, such as interviews or focus groups. One benefit of this design is that some fathers may have taken part who may have otherwise chosen not to do so. However, it is also a limitation, as free-text responses to open-ended questions do not provide the same rich data as other forms of qualitative research, such as interviews. It also means that it was not possible to ask any follow-up questions or ask participants to share more details. It is important that future research utilises a range of designs, including interviews and focus groups, to allow the field to access rich data, as well as the views of harder-to-reach fathers.

Finally, due to the focus of this research being on psychological help-seeking, it is possible that other forms of help-seeking, such as informal support or medication, were missed. Although research suggests that fathers prefer to seek psychological support [44], the qualitative data highlighted a desire to build informal, supportive connections through social and community groups. Therefore, focusing solely on psychological help-seeking may have missed between-group differences in intentions or access to other types of support. Future research should define help-seeking more precisely and consider collecting data on informal support and medication use.

## 5. Conclusions

This research suggests that fathers with PPD experience many of the same barriers to accessing psychological support as men with depression occurring at another stage of life. These include conformity to masculine stereotypes, a lack of awareness of paternal perinatal depression, exclusion from services, and self-stigma. Developing interventions which aim to reduce these barriers at the personal and public level should be a key target in encouraging more fathers to access the support they require for their mental health. Additionally, future research with larger, representative samples is required to better understand the barriers fathers with PPD face to seeking help.

## Figures and Tables

**Table 1:** Demographic data comparing the Father Depression (FD) group and the Male Depression (MD) group.

Demographic Variable	Father Depression (FD) *n* = 64	Male Depression (MD) *n* = 61	*p*-Value
Age	*n* = 64	*n* = 61	<0.001 **
18–25 years	3	22	
26–35 years	34	20	
36–55 years	27	16	
56+ years	0	3	
Ethnicity	*n* = 64	*n* = 61	0.74
White British	52	51	
Black British	1	0	
Asian British	2	1	
White Non-British	7	5	
Black Non-British	0	1	
Asian Non-British	1	0	
Other	1	3	
Father status	*n* = 64	*n* = 61	<0.001 **
Father	64	18	
Not father	0	43	
Total number of children	*n* = 64	*n* = 18 (fathers only)	0.64
1	33	7	
2	24	9	
3	5	1	
4+	2	1	
Time Since Birth of Youngest Child (years)	*n* = 62	*n* = 18 (fathers only)	^a^
Mean (range)	1.07 (0.05–1.95)	9.85 (2.79–31.36)	

** indicates *p* < 0.001. ^a^—it was not possible to statistically compare means due to the variation in distribution between the groups.

**Table 2:** Means, standard deviations and significance values for the between-subjects comparisons.

Measure	Father Depression (FD) *n* = 64	Male Depression (MD) *n* = 61	*p*-Value
ATSPPH-SF	*n* = 64	*n* = 61	0.65
Mean (SD)	18.88 (4.45)	19.33 (6.44)	
SSSHS	*n* = 59	*n* = 58	0.27
Mean (SD)	25.76 (5.74)	24.45 (7.21)	
CMNI-46	*n* = 53	*n* = 56	0.11
Mean (SD)	51.38 (9.46)	55.04 (13.74)	
Awareness of services	*n* = 53	*n* = 57	0.18
Median	3	3	
DASS-21	*n* = 62	*n* = 58	
Mean Depression (SD)	22.13 (9.96)	24.24 (12.72)	0.32
Mean Anxiety (SD)	11.74 (8.42)	13.31 (10.29)	0.36
Mean Stress (SD)	25.39 (9.56)	22.14 (9.75)	0.07
Mean Total (SD)	59.26 (24.95)	59.69 (28.73)	0.93
History of Depression	*n* = 60	*n* = 57	0.96
Yes	45	43	
No	15	14	
Partner Current Depression	*n* = 58 ^b^	*n* = 37	0.73
Yes	16	9	
No	42	28	
History of Seeking Help	*n* = 60	*n* = 58	0.71
Yes	31	28	
No	29	30	
Currently Seeking Help	*n* = 60	*n* = 58	0.84
Yes	17	18	
No	43	40	

^b^—group sizes are lower as one man in the FD group and 18 men in the MD group indicated they did not have a partner. Attitudes Towards Seeking Professional Psychological Help Scale short form (ATSPPH-SF); Self-Stigma of Seeking Help Scale (SSSHS); The Conformity to Masculine Norms Inventory-46 (CMNI-46); Depression, Anxiety and Stress Scale (DASS-21).

**Table 3:** Details of regression analysis and final model. *n* = 52. The dependent variable used in the analysis was psychological help-seeking intentions.

Predictor Variable(s) Included at Each Stage of Regression Analysis	R^2^	Adj R^2^	R^2^ Change	Beta	Standardised Beta	t	*p*
Conformity to masc. norms Awareness of Services	0.033	−0.007	0.033	−0.03 0.38	−0.07 0.12	−0.54 0.91	0.59 0.37
Self-stigma of seeking help	0.183	0.132	0.151	−0.29	−0.41	−2.99	0.00
Partner depression	0.184	0.114	0.001	0.06	0.01	0.05	0.96
Current depression	0.223	0.139	0.040	0.04	0.21	1.53	0.13

## Data Availability

The research data is not publicly available due to privacy or ethical restrictions.
